# Combination of pre-treatment dynamic [^18^F]FET PET radiomics and conventional clinical parameters for the survival stratification in patients with IDH-wildtype glioblastoma

**DOI:** 10.1007/s00259-022-05988-2

**Published:** 2022-10-13

**Authors:** Zhicong Li, Adrien Holzgreve, Lena M. Unterrainer, Viktoria C. Ruf, Stefanie Quach, Laura M. Bartos, Bogdana Suchorska, Maximilian Niyazi, Vera Wenter, Jochen Herms, Peter Bartenstein, Joerg-Christian Tonn, Marcus Unterrainer, Nathalie L. Albert, Lena Kaiser

**Affiliations:** 1 Department of Nuclear Medicine, University Hospital, LMU Munich, Marchioninistr. 15, 81377 Munich, Germany; 2 Center for Neuropathology and Prion Research, LMU Munich, Munich, Germany; 3 Department of Neurosurgery, University Hospital, LMU Munich, Munich, Germany; 4 Department of Neurosurgery, Sana Hospital, Duisburg, Germany; 5 Department of Radiotherapy, University Hospital, LMU Munich, Munich, Germany; 6 German Cancer Consortium (DKTK), Partner Site Munich, German Cancer Research Center (DKFZ), Heidelberg, Germany; 7 Department of Radiology, University Hospital, LMU Munich, Munich, Germany

**Keywords:** Radiomics, [^18^F]FET PET, Survival, Glioma

## Abstract

**Purpose:**

The aim of this study was to build and evaluate a prediction model which incorporates clinical parameters and radiomic features extracted from static as well as dynamic [^18^F]FET PET for the survival stratification in patients with newly diagnosed IDH-wildtype glioblastoma.

**Methods:**

A total of 141 patients with newly diagnosed IDH-wildtype glioblastoma and dynamic [^18^F]FET PET prior to surgical intervention were included. Patients with a survival time ≤ 12 months were classified as short-term survivors. First order, shape, and texture radiomic features were extracted from pre-treatment static (tumor-to-background ratio; TBR) and dynamic (time-to-peak; TTP) images, respectively, and randomly divided into a training (*n* = 99) and a testing cohort (*n* = 42). After feature normalization, recursive feature elimination was applied for feature selection using 5-fold cross-validation on the training cohort, and a machine learning model was constructed to compare radiomic models and combined clinical-radiomic models with selected radiomic features and clinical parameters. The area under the ROC curve (AUC), accuracy, sensitivity, specificity, and positive and negative predictive values were calculated to assess the predictive performance for identifying short-term survivors in both the training and testing cohort.

**Results:**

A combined clinical-radiomic model comprising six clinical parameters and six selected dynamic radiomic features achieved highest predictability of short-term survival with an AUC of 0.74 (95% confidence interval, 0.60–0.88) in the independent testing cohort.

**Conclusions:**

This study successfully built and evaluated prediction models using [^18^F]FET PET-based radiomic features and clinical parameters for the individualized assessment of short-term survival in patients with a newly diagnosed IDH-wildtype glioblastoma. The combination of both clinical parameters and dynamic [^18^F]FET PET–based radiomic features reached highest accuracy in identifying patients at risk. Although the achieved accuracy level remained moderate, our data shows that the integration of dynamic [^18^F]FET PET radiomic data into clinical prediction models may improve patient stratification beyond established prognostic markers.

**Supplementary Information:**

The online version contains supplementary material available at 10.1007/s00259-022-05988-2.

## Introduction

The inclusion of mandatory molecular markers for diagnosis in the World Health Organization (WHO) Classification of Tumors of the Central Nervous System (CNS) in 2016 and revised in 2021 has led to a more rigid definition of prognostically distinct entities [1, 2]. In particular, the isocitrate dehydrogenase (IDH)-wildtype status is associated with a worse prognosis in adult diffuse astrocytic gliomas [3] and results in the diagnosis of a glioblastoma, WHO grade 4, according to the 2021 WHO classification. Additional predictive markers such as the methylation status of the O-6-methylguanine-DNA-methyltransferase (MGMT) promotor further help to stratify brain tumor patients according to their individual risk profile [4]. However, even within the distinct molecularly defined tumor type of IDH-wildtype glioblastomas, few patients survive several years whereas others remain short-term survivors (STS) and decease within the first year, indicating further potential for improvement regarding patient stratification [5]. Balancing aggressive treatment including radiation and chemotherapy with quality of life is critical for patients [6].Therefore, additional prognostic markers beyond established molecular genetic markers and a stratification of survival beyond the neuropathological classification of brain tumors would be helpful to further improve individual prognostication and guide patient management accordingly.

Molecular imaging using positron emission tomography (PET) with radiolabeled amino acids such as *O*-(2-[^18^F]-fluoroethyl)-L-tyrosine ([^18^F]FET) has been applied successfully for the characterization and evaluation of primary brain neoplasms [7–9]. Hence, PET imaging was recommended by the Response Assessment in Neuro-Oncology (RANO) Working Group as useful imaging method in addition to conventional magnetic resonance imaging (MRI) in the clinical management of brain tumor patients [10]. Especially dynamic [^18^F]FET PET has been shown to be helpful for non-invasive tumor classification [11] and for individual prognostication even within defined molecular subgroups [7, 12]. Here, radiomics have recently gained increasing interest as a promising non-invasive tool, where quantitative features are extracted from medical images and combined with clinical and genomic information to establish predictive models [13, 14]. However, up to now, there is no radiomic approach based on dynamic [^18^F]FET PET data which aims to perform survival stratification specifically in patients with an IDH-wildtype glioblastoma, despite being one of the most common and aggressive brain tumors.

Therefore, the purpose of this study was to build and evaluate a prediction model, which incorporates clinical parameters and radiomic features extracted from static as well as dynamic [^18^F]FET PET for an individualized survival stratification in patients with a newly diagnosed IDH-wildtype glioblastoma.

## Materials and methods

### Patients

The retrospective analysis of PET imaging and clinical data was approved by the institutional review board of the LMU Munich (604–16), and all patients gave written informed consent before the PET scan. Patients with primary diagnosis of a glioma who received a pre-treatment dynamic [^18^F]FET PET scan at the Department of Nuclear Medicine of the LMU Munich were identified for this retrospective study. The inclusion criteria for analysis were (1) histologically confirmed IDH-wildtype glioblastoma according to the updated 2016 WHO classification [1]; (2) pre-treatment evaluation of a dynamic [^18^F]FET PET scan (ECAT EXACT HR + , Siemens Healthineers, Inc., Erlangen, Germany; Siemens Medical Systems, Inc., Erlangen, Germany); (3) [^18^F]FET-positive glioma (tumor-to-background ratio, TBR ≥ 1.6); and (4) availability of clinical characteristics, including age, gender, Karnofsky Performance Score (KPS), as well as MGMT promoter methylation status and telomerase reverse transcriptase promoter (TERTp) mutation status. Patients with no follow-up data were excluded. Patients with a survival time ≤ 12 months were defined as short-term survivors (STS) [15, 16].

### [^18^F]FET PET image acquisition

[^18^F]FET PET images were acquired on an ECAT EXACT HR + PET scanner (Siemens Healthineers) with the standard protocol [8, 17] at the Department of Nuclear Medicine of the LMU Munich. Dynamic [^18^F]FET PET images were acquired over 40 as detailed in [14]. If relevant motion was observed in dynamic PET images, a frame-wise correction was performed using PMOD fusion tool (version 3.5; PMOD Technologies, Zurich, Switzerland) after frame-wise checking for motion.

### Segmentation of tumor volumes and brain background

The mean background activity was assessed from a large crescent-shaped volume of interest (VOI) in the contralateral healthy hemisphere as published previously [18] and recommended in the Joint EANM/EANO/RANO practice guidelines/SNMMI procedure standards for imaging of gliomas using PET with radiolabeled amino acids [19]. For tumor segmentation, a VOI was delineated with a TBR-threshold of 1.6 in static 20–40 min p.i. summation images as previously described [20].

### TBR and TTP image generation

The image values were normalized with the mean background value to generate static 20–40 min p.i. (TBR_20–40_) TBR images. An in-house developed software described previously by Kaiser et al. [21] (C +  + with integration of the ROOT data analysis framework, version 6.22/08, CERN, Switzerland; and ITK segmentation and registration toolkit 4.13.3, National Library of Medicine, National Institutes of Health, USA) was applied to generate voxel-wise parametric images. For the generation of TTP images, time–activity curves (TACs) were derived from each voxel, which were then classified according to the time frame reaching the peak uptake, i.e., (1) < 5 min, (2) 5–10 min, (3) 10–15 min, (4) 15–20 min, (5) 20–30 min, and (6) 30–40 min. TTP analyses excluded the first 2.7 min p.i. to avoid influence from early blood flush [21]. In case of a positive late slope (15–40 min p.i.), the TTP was assigned to group 6.

### Radiomic feature extraction

Images were resampled to isotropic voxels using linear interpolation (size 2.03 × 2.03 × 2.03 mm^3^), then radiomic features were extracted in Python (version 3.8.5) using PyRadiomics (version 3.0.1) [22], which complies with the Imaging Biomarker Standardization Initiative (IBSI) guidelines [23]. The included feature classes were first-order features, shape features and texture features, which were extracted from TBR and TTP images, respectively. No image filters were applied. As previously published, a fixed intensity bin size was set to 0.13 for TBR_20–40_ images, resulting from the average interquartile range divided by 4 [21, 24, 25]. The smallest time frame duration considered in the TTP categories was 5 min, which was used as the fixed bin width for feature extraction from TTP images.

### Machine learning pipeline

**Fig. 1:**
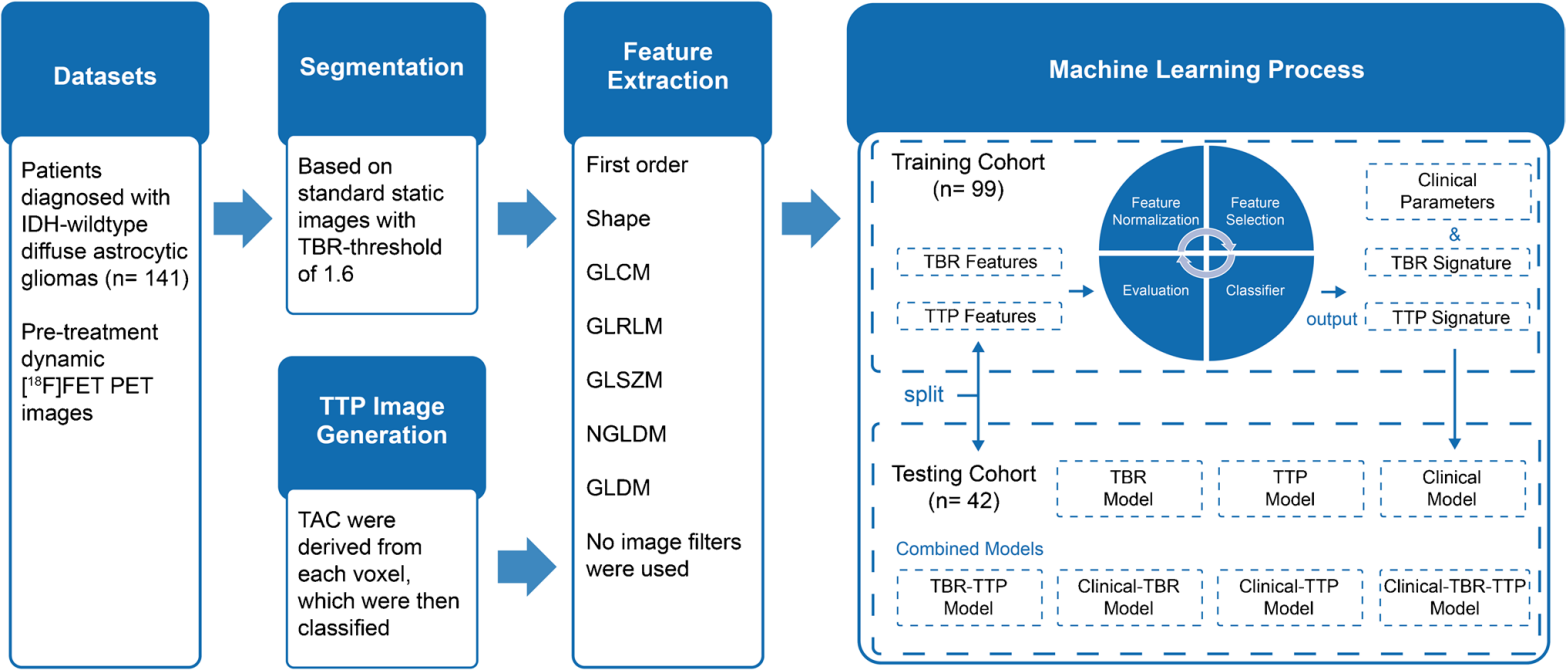
The workflow of radiomic process. TBR, tumor-to-background ratio; TTP, time-to-peak; TAC, time–activity curves; GLCM, gray level co-occurrence matrix; GLRLM, gray level run length; GLSZM, gray level size-zone matrix; NGLDM, neighborhood gray level different matrix; GLDM, gray level dependence matrix

Before feature selection, a stratified random split was used to assign 70% of the patients to the training cohort (*n* = 99) and the remaining 30% to the testing cohort (*n* = 42), with a balanced distribution of STS and non-STS (*P* = 0.8654, Pearson’s χ^2^ test) and clinical parameters in both groups using the FeAture Explorer (FAE) [26]. The independent testing cohort was not involved in the process of model training and used only for model testing. Machine learning including feature selection and model construction was implemented in Python (version 3.8.5) using scikit-learn package (version 0.24.1) [27]. The workflow of the processing pipeline is presented in Fig. 1.

Feature standardization was computed only on the training cohort and then applied to both the training and the testing cohorts. For each feature, the mean value and the standard deviation were calculated. The mean value was subtracted from each individual value, which was then divided by the standard deviation.

Before performance evaluation on the test set, feature selection and model fitting was conducted on the training set. Logistic regression (LR) models were built to predict short-term survival of GBM patients in the testing cohort by fitting selected features on the training cohort. For survival classification, LR was applied in “balanced” mode, which gives higher weight to the minority class and lower weight to the majority class. With this setting, weights are automatically adjusted inversely proportional to class frequencies in the input data to avoid the influence from the imbalance of comparison groups [28]. Considering the small amount of data, the solver “liblinear” was used and the maximum number of iterations was set to 1000 for the solver to converge. The remaining settings of the logistic regression classifier provided within the scikit-learn package were set to default.

### Radiomic feature selection

Pearson correlation coefficient (PCC) was used to reduce the dimensions of the feature matrix [29]. The PCC of two features was compared iteratively. If the PCC was larger than 0.99 [30], the second feature was removed. Furthermore, recursive feature elimination (RFE) based on logistic regression classifier was performed to reduce the number of redundant features and select potential survival-related features [31]. During each iteration, a feature which is considered least important is deleted. The number of features to select was chosen to range between 1 and 15. The performance of each model with a different number of features was assessed using the area under the receiver operating characteristic curve (AUC) obtained from repeated stratified cross-validation using three splits and five folds.

### Model construction and testing

First, models considering radiomic features derived from either TBR or TTP images or only clinical parameters were generated and compared to each other. Radiomic signatures were generated by using linear combinations of the selected radiomic features according to the LR coefficients in the TBR and TTP models. The clinical model was constructed from all clinical parameters including age, gender, KPS, MGMT promoter methylation status, and TERTp mutation status. Second, the TBR-TTP model was built from a combination of the TBR signature and the TTP signature. The combined clinical-radiomic models were constructed by combining clinical parameters and radiomic signatures, respectively.

### Statistical analysis

Receiver operating characteristic curve (ROC) analysis was performed on the training and testing cohorts to evaluate the model performance. AUC, accuracy, sensitivity, specificity, positive predictive value (PPV), and negative predictive value (NPV) were calculated for diagnostic power when applying the trained model on the testing cohort. Then, 95% confidence intervals (CIs) were calculated by using a non-parametric bootstrap method, which was repeated 1000 times to get a bootstrap distribution of the results.

Categorical variables or continuous variables were reported as numbers and percentages or as mean and standard deviation. Categorical variables were compared using Pearson’s χ^2^ test and continuous variables were compared using Mann–Whitney *U* test. *P* values < 0.05 were considered statistically significant.

Statistical analyses were implemented in Python (version 3.8.5) using scikit-learn package (version 0.24.1) [27].

## Results

### Patient characteristics

A total of 141 patients (median age, 59.3 years; range, 19.0–77.2 years) were included in this study. Of the 141 patients, 94 (66.7%) patients underwent stereotactic biopsy and 47 (33.3%) microsurgical resection at initial diagnosis, with the same distribution between the training and testing cohorts and no significant differences between both STS and non-STS group (*P* value = 0.355). Forty patients (28.4%) had a survival time of less than 12 months and were classified as STS. The variables which constructed the clinical model included age, gender, Karnofsky Performance Score, CNS WHO grade, MGMT promoter methylation status, and TERTp mutation status, and are presented in Table 1. There were no significant differences between the training and testing cohorts with regard to clinical parameters, with STS rates of 28.3% and 28.6%, respectively. The initial therapies of STS and non-STS are shown in Table S1.

**Table 1:** Clinical characteristics of the patients

	Training cohort (*n* = 99)		Testing cohort (*n* = 42)		*P* value
	STS	Non-STS	STS	Non-STS	
Characteristic	(*n* = 28)	(*n* = 71)	(*n* = 12)	(*n* = 30)	0.865
Age, years	56.7 ± 11.8		58.5 ± 13.1		0.121
Gender					
Female (0)	40 (40.4%)		17 (40.5%)		0.857
Male (1)	59 (59.6%)		25 (59.4%)		
KPS	80 (60–100)		80 (40–100)		0.587
WHO grade					
III	32 (32.3%)		16 (38.1%)		0.640
IV	67 (67.7%)		26 (61.9%)		
MGMT					
Unmethyl. (0)	47 (53.0%)		20 (51.2%)		0.988
Methyl. (1)	52 (47.0%)		22 (48.8%)		
TERTp					
Wildtype (0)	21 (21.2%)		10 (23.8%)		0.516
Mutation (1)	78 (78.8%)		32 (76.2%)		

Data are means ± standard deviations or numbers of patients with percentages in parentheses. *P* value was derived from the univariate association analyses between each clinical parameter. Calculated by using the Mann–Whitney *U* test for continuous variables and Pearson’s χ^2^ test for categoric variables. Gender, MGMT, TERTp with representative number of formula of risk probability in parentheses

*STS* short-term survivors, *KPS* Karnofsky Performance Score

### Radiomic feature extraction and selection

**Fig. 2:**
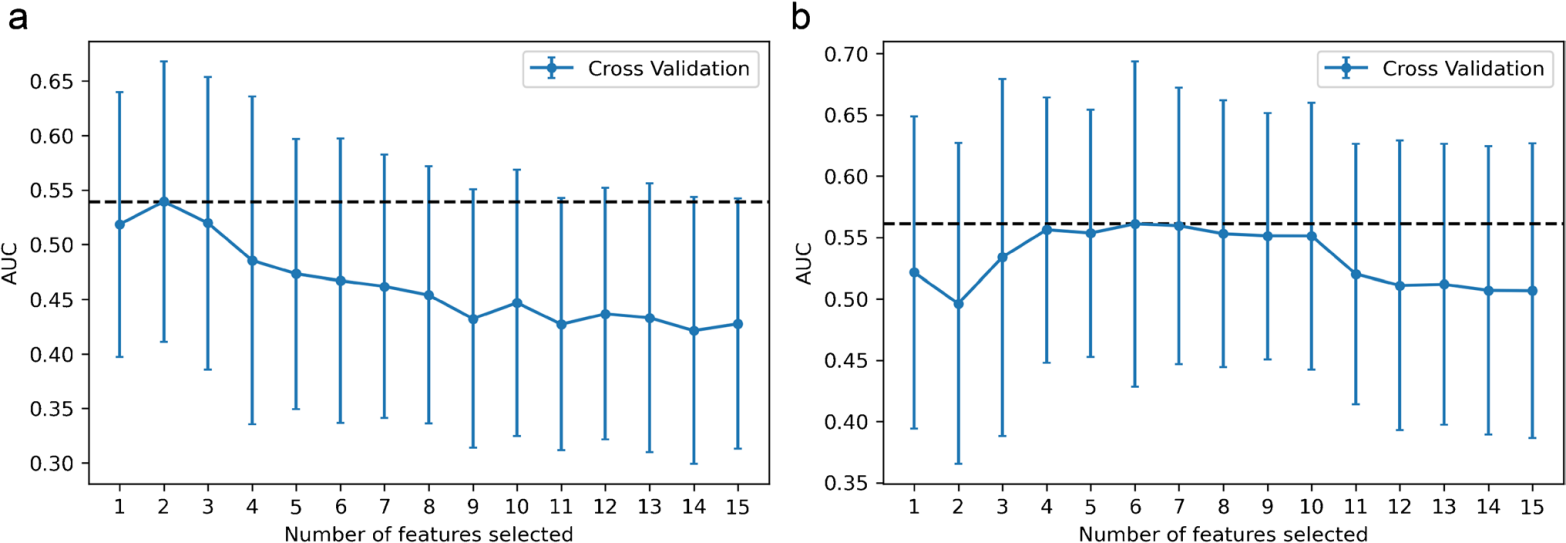
The feature selection process of the RFE. Each iteration removes a feature that is considered least important and corresponds to a 3-repeated 5-fold cross-validation. After cross-validation, the average AUC of the model in the training cohort was used to determine the optimal number of features. The number of candidate features was chosen to range from 1 to 15. The feature number with maximal AUC was selected. **a** Two features were selected in the TBR model and **b** six features were selected in the TTP model. RFE, recursive feature elimination; AUC, area under the receiver operating characteristic curve

The original features considered for the model construction included six clinical parameters and 107 radiomic features extracted from static and dynamic [^18^F]FET PET images, respectively. After the PCC-based exclusion of redundant features, 79 features were retained from TBR images and 94 features were retained from TTP images. With RFE, two features were finally selected for the TBR model and six features for the TTP model (Fig. 2).

### Diagnostic validation of the TBR model, TTP model, and clinical model

The TBR model reached an AUC of 0.63 (95% CI, 0.52–0.75) in the training cohort for the prediction of STS (Supplementary Fig. S1a, S1b), with a sensitivity of 60.7% and a specificity of 60.6%, and a similar AUC of 0.63 (95% CI, 0.47–0.78) in the testing cohort, with a sensitivity of 50.0% and a specificity of 73.3%. The TTP model showed a higher predictability of STS (Fig. S1c, S1d) with an AUC of 0.77 (95% CI, 0.69–0.84) in the training cohort (sensitivity 75.0% and specificity 63.4%), and with an AUC of 0.71 (95% CI, 0.57–0.84) in the testing cohort (sensitivity 50.0% and specificity 70.0%). The clinical model demonstrated an accuracy at a comparable level as the TTP model (Fig. S1e, S1f), with an AUC of 0.79 (95% CI, 0.71–0.86) in the training cohort (sensitivity 75.0% and specificity 64.8%) and an AUC of 0.69 (95% CI, 0.50–0.86) in the testing cohort (sensitivity 66.7% and specificity 53.3%).

The coefficients of features in the clinical model are shown in Supplementary Table S2. Radiomic signatures are provided in Supplementary section S2. Detailed information about the performance of the different models is shown in Table 2 and Supplementary Table S3.

**Table 2:** Performance of TBR, TTP, and clinical models for the testing cohort

	TBR model	TTP model	Clinical model
AUC	0.63	0.71	0.69
AUC 95% CI	(0.47–0.78)	(0.57–0.84)	(0.50–0.86)
Accuracy	66.7%	64.3%	57.1%
Sensitivity	50.0%	50.0%	66.7%
Specificity	73.3%	70.0%	53.3%
PPV	42.9%	40.0%	36.4%
NPV	78.6%	77.8%	80.0%

*CI* confidence interval, *TBR* tumor-to-background ratio, *TTP* time-to-peak

### Diagnostic validation of the combination models

The combined TBR-TTP model reached an AUC of 0.79 (95% CI, 0.72–0.87) in the training cohort for the prediction of STS (Supplementary Fig. S2a, S2b), with a sensitivity of 71.4% and a specificity of 69.0%, and an AUC of 0.74 (95% CI, 0.61–0.86) in the testing cohort, with a sensitivity of 50.0% and a specificity of 70.0%.

The combined clinical-TBR model showed only slightly higher predictability of STS than the TBR model, with an AUC of 0.80 (95% CI, 0.72–0.87) in the training cohort and 0.64 (95% CI, 0.47–0.81) in the testing cohort (Fig. S2c, S2d). The sensitivity and specificity were 75.0% and 70.4% in the training cohort, and 58.3% and 60.0% in the testing cohort, respectively.

The combined clinical-TTP model showed best predictability of STS, with an AUC of 0.86 (95% CI, 0.78–0.92) in the training cohort (sensitivity 82.1% and specificity 74.7%) and 0.74 (95% CI, 0.60–0.88) in the testing cohort (sensitivity 66.7% and specificity 70.0%) (Fig. S2e, S2f).

The clinical-TBR-TTP model reached an AUC of 0.86 (95% CI, 0.70–0.93) in the training cohort for the prediction of STS (Fig. S2g, S2h), with a sensitivity of 89.3% and a specificity of 71.8%, and AUC of 0.72 (95% CI, 0.59–0.86) in the testing cohort, with a sensitivity of 58.3% and a specificity of 73.3%.

LR coefficients of the combined models are provided in Supplementary section S3. Detailed information about the performance of the combined models is shown in Table 3 and Supplementary Table S4.

**Table 3:** Performance of combined models for the testing cohort

Model	AUC	95% CI	Accuracy (%)	Sensitivity (%)	Specificity (%)	PPV (%)	NPV (%)
TBR-TTP	0.74	(0.61–0.86)	64.3	50.0	70.0	40.0	77.8
Clinical-TBR	0.64	(0.47–0.81)	59.5	58.3	60.0	36.8	78.3
Clinical-TTP	0.74	(0.60–0.88)	69.0	66.7	70.0	47.1	84.0
Clinical-TBR-TTP	0.72	(0.59–0.86)	69.0	58.3	73.3	46.7	81.5

*CI* confidence interval, *TBR* tumor-to-background ratio, *TTP* time-to-peak

## Discussion

This study illustrates that integration of radiomics based on dynamic [^18^F]FET PET may improve the assessment of short-term survival probability in patients with newly diagnosed IDH-wildtype glioblastoma. As opposed to prediction models based on clinical parameters or radiomic features alone, specifically a combined clinical-TTP model including both clinical parameters and an additional radiomic signature derived from dynamic PET accomplished a higher prognostic value for short-term survival.

Several studies have analyzed the role of [^18^F]FET PET for the assessment of survival probability in patients with glioma [7, 8, 12, 32–35]. It has been reported that a large biological tumor volume (BTV) on static [^18^F]FET PET [32, 33, 35] as well as a short TTP_min_ extracted from dynamic [^18^F]FET PET at initial diagnosis are associated with STS [7, 12, 34, 35]. Besides, Bauer et al. showed that TTP_min_ is an independent prognostic factor for overall survival, reaffirming the value of dynamic [^18^F]FET PET in the prediction of survival in glioma patients. Yet, initial radiomics data in high-grade glioma have been provided by MRI studies, achieving high AUC values for the prognostication of overall survival in the range of 0.652–0.858 in the test cohort [36–40] demonstrating that radiomics might be a valuable tool to estimate survival in brain tumor patients. Meanwhile, first promising studies have brought [^18^F]FET PET–based radiomics into the focus: Radiomic features extracted from static [^18^F]FET PET showed better accuracy than conventional static parameters (e.g., TBR_max_) to identify pseudoprogression [13]. For the differentiation between radiation injury and recurrence of brain metastasis, textural features extracted from [^18^F]FET PET had a diagnostic accuracy of 83% [41]. Carles et al. reported that [^18^F]FET PET radiomics could contribute to the prognostic assessment [42], and Paprottka et al. established a promising tool for objective differentiation of tumor progression from treatment-related changes by combining [^18^F]FET PET and multiparametric MRI [43]. However, those initial studies only analyzed static [^18^F]FET PET features without taking into account important clinical parameters and, furthermore, no study so far has utilized dynamic [^18^F]FET PET–based radiomics to assess the probability of poor prognosis within distinct molecular brain tumor types.

The present study used clinical parameters combined with [^18^F]FET PET radiomic features to develop combined clinical-radiomic models. A model based on clinical data only, built from six important survival-related clinical parameters, achieved an AUC of 0.69 in the independent testing cohort. A TBR model, built from two static [^18^F]FET PET features, achieved an AUC of 0.63 in the testing cohort and thus did not perform better than the clinical model. The TTP model, however, generated from six dynamic [^18^F]FET PET features, achieved an AUC of 0.71 in the testing cohort, thus slightly exceeding the clinical-only model and outranging the TBR-only model, highlighting the importance of dynamic PET data in the context of survival-related analyses. The combined purely imaging-based TBR-TTP model achieved only slightly better results than each model alone (AUC of 0.74 vs. AUC of 0.63 and AUC of 0.71). Eventually, the merger of the TTP radiomic signature and clinical data, resulting in the combined clinical-TTP model, achieved best predictive performance with an AUC of 0.74. Integrated discrimination improvement (IDI) was calculated between the clinical model and the combined clinical-TTP model [44]. The value of IDI was 0.1089, which was greater than 0, and the *P* value was 0.023, which was statistically significant. It indicated that the combination of TTP radiomics and clinical data, compared to clinical parameters alone, led to an improved ability of the model to identify patients at risk. Although intriguing to speculate that the clinical-TBR-TTP model would achieve highest accuracy as it includes all available information, the AUC did not improve, which may be related to the limited value of TBR information in this context, but this should be re-evaluated in larger cohorts. Taken together, as previously shown for other entities, it seems beneficial not to narrow the view to the clinical information alone when constructing a predictive model but to include radiomic signatures in clinical prediction studies as well, as the combination of clinical and radiomic information seems to be of particular value with regard to survival risk prediction [45]. When considered on its own, an AUC of 0.74 still does not seem satisfactory, as further underscored by a positive predictive value for the identification of a short-term survivor of only 47.1% even for the best model (see Table 3). From a clinical point of view, the positive and negative predictive values are highly useful metrics in the context of decision-making as they give an estimate on the correctness of a prediction. In the clinical setting, it would be particularly beneficial to identify patients at risk for short-term survival in order to facilitate the selection of more aggressive treatments or earlier inclusion in experimental treatment studies, rather than just standard treatment, to which approximately 30% of patients do not respond. However, also the identification of long-term survivors would be helpful in the clinical routine, as pseudoprogression can occur in one-third of the patients and may, when misinterpreted as tumor progression on MRI, lead to a premature cessation of an effective treatment. Of note, while the positive predictive value was extremely low in all models, the negative predictive value, reflecting the predictability of long-term survival, reached 84% in the best model. Therefore, even though the overall accuracies of our prediction models may not yet be satisfactory for the clinical use and the low positive predictive values impede the prediction of a short-term survivor, the high negative predictive value may be helpful for clinical decision-making. Our study supports that within a neuropathologically homogenous group of aggressive IDH-wildtype glioblastomas, especially the combination of different types of information (in this case clinical data and radiomic signature) can add value to a survival prediction model and consequently hints to the potential, which lies in the inclusion of even further image-based information. Indeed, one might speculate that the addition of conventional MRI data and in a next step more sophisticated MRI data such as perfusion or diffusion-weighted MRI may further increase the power of survival risk prediction of the combined clinical-TTP model [46], but such analyses require a standardized imaging protocol to assure comparability of MRI-based radiomic features. In other tumor entities as well, especially multiparametric imaging approaches have shown highly promising results for survival prediction, e.g., reaching an accuracy of up to 98% in a study on cervical cancer as compared to only 56–60% for prediction models using the standard clinical variables alone [47, 48]. Accordingly, dual PET imaging studies including other tracers than [^18^F]FET in IDH-wildtype glioblastoma, such as TSPO-ligands which offer complementary information to the [^18^F]FET uptake [49], are of high potential to further increase the power of survival prediction models, as exemplified by recent successful multi-tracer PET prediction approaches in other entities, such as prostate cancer [50].

Although the number of patients included in the current study is by far higher than in most previous [^18^F]FET PET radiomics studies, a further increase in patient numbers may in future result in outperforming radiomics-only based approaches, as already shown in large-scale analyses for other medical settings [51]. According to the above-generated multivariate LR-based formulas, the known risk factors of high WHO grade, unmethylated MGMT promoter, TERTp mutation as well as higher patient age and lower KPS at diagnosis of IDH-wildtype glioblastoma were more likely associated with short-term survival [52–55]. However, gender has different correlations in different formulas, which is inconsistent with the literature [53], although the weight of this parameter was low. This may likewise be due to the relatively low number of patients included in this study.

Whereas, in clinical routine, established dynamic [^18^F]FET PET parameters such as the time–activity curve and/or the slope are usually only derived from representative subvolumes of interest within the tumor [7, 9, 56], in the current study every single voxel of the tumor was analyzed in order to generate whole-tumor TTP maps of dynamic [^18^F]FET PET images. This comprehensive whole-tumor approach facilitated radiomic features extraction in dynamic image data and ensured to account for heterogeneity of uptake kinetics which has a major clinical impact when assessing brain tumors in dynamic [^18^F]FET PET [57]. In this context, a relationship between tumor heterogeneity and the STS group could be found in the feature *ClusterProminence (CP)*. CP belongs to the *Gray Level Co-occurrence Matrix (GLCM)* and measures the skewness and asymmetry of the GLCM. A higher value implies more asymmetry while a lower value indicates a peak near the mean value and less variation around the mean. This correlation with the STS group indicates that a patient with a heterogeneous tumor in dynamic [^18^F]FET PET images is more likely to be identified as high-risk patient for short-term survival. Another exemplary radiomic feature, which is associated with the STS group, is *Maximum 3D diameter*, *3D shape feature*. The latter is defined as the largest pairwise Euclidean distance between tumor surface mesh vertices. This correlation, in simplified terms, indicates that patients belonging to the STS group have a tumor that shows large spread on PET. This finding is consistent with the literature—large tumor volumes on [^18^F]FET PET were reported to be associated with poor overall survival in glioblastoma patients before radiation therapy with concomitant and adjuvant temozolomide [32, 33]. Details of other features are shown in the Supplementary information.

There are several limitations to this study. Only single-center data have been investigated, which led to the relatively small sample size and the lack of external validation. Yet, only single-center data have been chosen in this study since dynamic [^18^F]FET PET is not always acquired routinely in other centers and pooling PET data with differences in time framing, image reconstruction algorithm, and scanner type may require prior implementation and validation of, e.g., feature harmonization procedures [58]. Moreover, it should be noted that almost all previous [^18^F]FET PET radiomics studies have been performed with much smaller numbers of cases. The reliability of the reported scores was additionally evaluated using nested cross-validation [59] with five random splits in the outer loop, yielding a high AUC variability of 10% for the TTP model, 15% for the TBR model, and 11% for the clinical model (Supplementary material S4). Thereby, different radiomic signatures were obtained for each split of the outer loop since feature selection and model building are not robust when dealing with small sample sizes. Feature selection represents a challenge and has an impact on the performance of prediction models. Other feature selection methods comprise, e.g., filter methods such as minimum redundancy maximum relevance (MRMR) or ensemble methods, which provide a good balance between robust feature selection and model performance. Wrapper methods such as RFE have the advantage that feature dependencies can be modeled and that they interact with the classifier, while also bearing the risk of overfitting [60]. To enable standardized segmentation of tumor regions, only positive [^18^F]FET PET images were included. Furthermore, MRI-based radiomics, as a more widely established and complementary tool, were not included in this study. Future studies may benefit from the combined use of multiparametric MRI data.

## Conclusion

This study built and evaluated prediction models for survival combining both radiomic features extracted from static and dynamic [^18^F]FET PET and clinical parameters. Specifically, the combination of clinical parameters with radiomics based on dynamic [^18^F]FET PET data achieved a higher prognostic accuracy for the individualized assessment of short-term survival in patients with newly diagnosed IDH-wildtype glioblastoma in comparison to models using conventional clinical parameters only. Although the final accuracy remained moderate, the integration of dynamic [^18^F]FET PET radiomic data into clinical prediction models may improve patient stratification beyond established prognostic markers. Future prospective radiomic studies using multimodal imaging data are needed to evaluate whether the integration of additional imaging parameters may further improve the prognostic performance and enhance the clinical interpretation of the study results.

## Supplementary Information

Below is the link to the electronic supplementary material.

Supplementary file1 (DOCX 1180 KB)

## Data Availability

The data that support the findings of this study are available on request from the corresponding author, ZL. The data are not publicly available due to the privacy of research participants.

## Code availability

The code that support the findings of this study are available from ZL and LK on reasonable request.
