# Supplementary material for: Combination of pre-treatment dynamic [18F]FET PET radiomics and conventional clinical parameters for the survival stratification in patients with IDH-wildtype glioblastoma
Source: Eur J Nucl Med Mol Imaging. 2022 Oct 13;50(2):535–45. doi: 10.1007/s00259-022-05988-2 (PMC9816231; doi:10.1007/s00259-022-05988-2)
Supplement: Supplementary file 1 — Supplementary file1 (DOCX 1180 KB) [file 259_2022_5988_MOESM1_ESM.docx]

**S1 Radiomics features**

In this study, 107 radiomics features of candidates were generated from dynamic ^18^F-FET PET images, including first-order statistics, shape-based and texture features. First order features describe the distribution of grey values in VOIs, such as range, entropy and variance. Shape-based features describe the three-dimensional size and shape of the VOIs, such as volume, surface area and maximum diameter. Texture features reflects the texture characteristics of tumor in terms of the interrelationship between pixels, including grey level co-occurrence matrix (GLCM), grey level run length matrix (GLRLM), grey level size-zone matrix (GLSZM), neighborhood grey level different matrix (NGLDM) and grey level dependence matrix (GLDM) features.

The detailed description of each feature can be found in the Pyradiomics documentation (https://pyradiomics.readthedocs.io/en/latest/).

**S2 Development of Radiomic Signatures from the TBR and TTP Model**

The radiomic signatures consisted of two radiomic features in the TBR model and six features in the TTP model, the formulas of radiomic signatures were as follows:

$$radiomic signature in the TBR model=Kurtosis\times0.764 +TotalEnergy\times(-0.686)-0.026$$

$$radiomic signature in the TTP model=Coarseness\times1.138 +SmallAreaHighGrayLevelEmphasis\times0.695 +ShortRunHighGrayLevelEmphasis\times0.649 +Maximum3DDiameter\times0.608 +SurfaceVolumeRatio\times\left( -0.537 \right)+ClusterProminence\times0.530 -0.265$$

**S3 Development of Combination Models**

In the TBR-TTP model, the formulas were as follows:

$$risk probability in the TBR-TTP model =TBR radiomic signature\times0.474+TTP radiomic signature \times1.222-0.328$$

In the combined clinical-radiomic model, besides the radiomic signatures, six clinical parameters were included as well. The formulas were as follows:

$$risk probability in the clinical-TBR model =radiomic signature\times0.399+Gender \times0.139+Age\times0.314+KPS\times\left( -0.166 \right)+WHO grade\times0.905+MGMT\times\left( -0.344 \right)+TERTp\times0.195-0.338$$

$$risk probability in the clinical-TTP model =radiomic signature\times1.118+Gender \times\left( -0.021 \right)+Age\times0.325+KPS\times\left( -0.409 \right)+WHO grade\times0.744+MGMT\times\left( -0.327 \right)+TERTp\times0.001-0.183$$

In the clinical-TBR-TTP model, combined with clinical parameters, TBR signature as well as TTP signature, the formulas were as follows:

$$risk probability in the clinical-TBR-TTP model =TTP radiomic signature\times1.091+TBR radiomic signature\times0.376+Gender \times0.008+Age\times0.230+KPS\times\left( -0.272 \right)+WHO grade\times0.788+MGMT\times\left( -0.279 \right)+TERTp\times0.048-0.527$$

The output of the model represents the risk probability for STS. In case of a risk probability > 0.5, the model classified the corresponding case as STS.

**S4 Evaluation of the variability of testing cohort in the TTP model**

To analyze the effect of testing set variability on the results, we randomly split the original data 5 times using FAE, which ensures that training and testing cohorts have a balanced distribution of clinical parameters. The results are shown in Table S5. The mean AUC in the testing cohort is 0.71 for TTP features, 0.54 for TBR features, and 0.65 for clinical parameters with standard deviations 0.07 (10%), 0.08 (15 %), and 0.07 (11 %) (Fig. S3).

**Tables**

**Table S1**: Differences between STS and non-STS: initial therapy

| Therapy | STS (n = 40) | non-STS (n = 101) |
| --- | --- | --- |
| Combined radiochemotherapy | 26 (65.0%) | 75 (74.2%) |
| Chemotherapy | 4 (10.0%) | 14 (13.9%) |
| Radiotherapy | 9 (22.5%) | 10 (9.9%) |
| Brachytherapy | 0 (0.0%) | 2 (2.0%) |
| None | 1 (2.5%) | 0 (0.0%) |
| *P* | 0.130 | |
| Stereotactic biopsy | 29 (72.5%) | 65 (64.4%) |
| Microsurgical resection | 11 (27.5%) | 36 (35.6%) |
| *P* | 0.355 | |

Calculated by using Fisher's exact test and Pearson’s χ^2^ test, respectively.

STS, short-term survivors.

^*^*P* < 0.05.

**Table S2**: Coefficients of features in the clinical model

| Features | Coefficients |
| --- | --- |
| Gender | 0.095 |
| Age | 0.400 |
| KPS | -0.313 |
| WHO grade | 0.852 |
| MGMT | -0.383 |
| TERTp | 0.1588 |

Note. — Intercept $\theta_{0}$ is -0.320 in the clinical model.

**Table S3**: Performance of TBR, TTP and Clinical models for the training cohort

|  | TBR Model | TTP Model | Clinical Model |
| --- | --- | --- | --- |
| AUC | 0.63 | 0.77 | 0.79 |
| AUC 95%CI | (0.52-0.75) | (0.69 - 0.84) | (0.71 - 0.86) |
| Accuracy | 60.6% | 66.7% | 67.7% |
| Sensitivity | 60.7% | 75.0% | 75.0% |
| Specificity | 60.6% | 63.4% | 64.8% |
| PPV | 37.8% | 44.7% | 45.7% |
| NPV | 79.6% | 86.5% | 86.8% |

CI, confidence interval; TBR, tumor-to-background ratio; TTP, time-to-peak.

**Table S4**: Performance of combined models for the training cohort

| Model | AUC | CI 95% | Accuracy | Sensitivity | Specificity | PPV | NPV |
| --- | --- | --- | --- | --- | --- | --- | --- |
| TBR-TTP | 0.79 | (0.72 - 0.87) | 69.7% | 71.4% | 69.0% | 47.6% | 86.0% |
| Clinical-TBR | 0.80 | (0.72 - 0.87) | 71.7% | 75.0% | 70.4% | 50.0% | 87.7% |
| Clinical-TTP | 0.86 | (0.78 - 0.92) | 76.8% | 82.1% | 74.6% | 56.1% | 91.4% |
| Clinical-TBR-TTP | 0.86 | (0.79 - 0.93) | 76.8% | 89.3% | 71.8% | 55.6% | 94.4% |

CI, confidence interval; TBR, tumor-to-background ratio; TTP, time-to-peak.

**Table 1:** The AUC results for different splits into training and testing cohort

| Case | Number of features in TTP model | Training cohort in TTP model | Testing cohort in TTP model | Number of features in TTP model | Training cohort in TBR model | Testing cohort in TBR model | Training cohort in clinical model | Testing cohort in clinical model |
| --- | --- | --- | --- | --- | --- | --- | --- | --- |
| Manuscript | 6 | 0.77 | 0.71 | 2 | 0.63 | 0.63 | 0.79 | 0.69 |
| 1 | 6 | 0.80 | 0.73 | 9 | 0.84 | 0.54 | 0.83 | 0.62 |
| 2 | 9 | 0.82 | 0.74 | 9 | 0.80 | 0.63 | 0.74 | 0.74 |
| 3 | 6 | 0.80 | 0.61 | 2 | 0.57 | 0.51 | 0.80 | 0.54 |
| 4 | 9 | 0.82 | 0.81 | 10 | 0.79 | 0.48 | 0.77 | 0.70 |
| 5 | 6 | 0.72 | 0.66 | 9 | 0.79 | 0.43 | 0.77 | 0.63 |

**Figures**


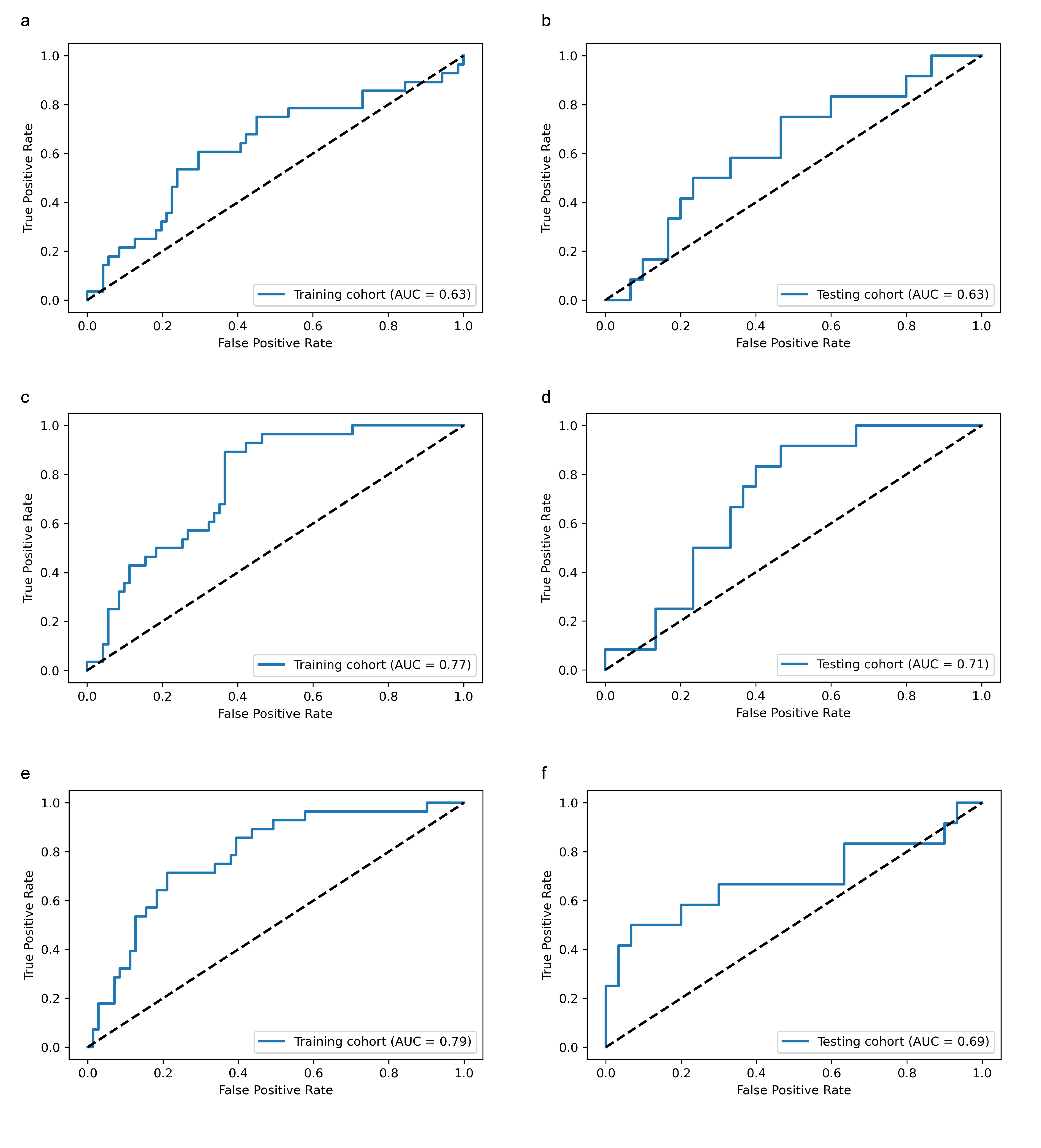


**Figure S1:** (a) The TBR model reached an AUC of 0.63 in the training cohort, and (b) an AUC of 0.63 in the testing cohort. (c) The TTP model reached an AUC of 0.77 in the training cohort, and (d) an AUC of 0.71 in the testing cohort. (e) The clinical model reached an AUC of 0.79 in the training cohort, and (f) an AUC of 0.69 in the testing cohort. AUC, area under the receiver operating characteristic curve.

**

Figure S2:** (a) The TBR-TTP model reached an AUC of 0.79 in the training cohort, and (b) an AUC of 0.74 in the testing cohort. (b) The clinical-TBR model reached an AUC of 0.80 in the training cohort, and (c) an AUC of 0.64 in the testing cohort. (e) The clinical-TTP model reached an AUC of 0.86 in the training cohort, and (f) an AUC of 0.74 in the testing cohort. (g) The clinical-TBR-TTP model reached an AUC of 0.86 in the training cohort, and (h) an AUC of 0.72 in the testing cohort. AUC, area under the receiver operating characteristic curve.


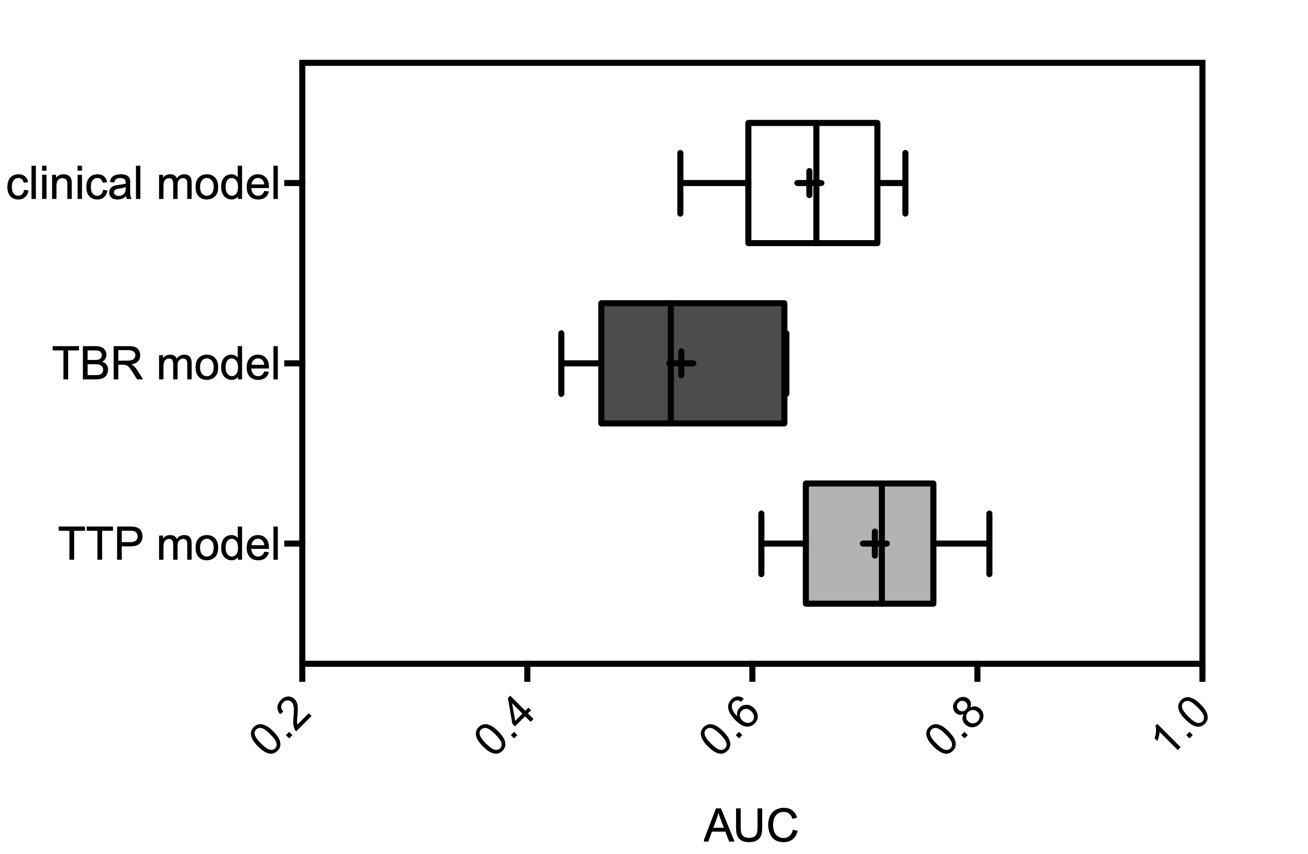


**Figure S3:** The AUC results of the testing cohort for different splits.
